# Analysis of Healthcare-associated Infections before and during the COVID-19 pandemic in a Colombian hospital

**DOI:** 10.15649/cuidarte.3624

**Published:** 2024-05-30

**Authors:** Luz M. Wintaco, Doris C. Quintero-Lesmes, José A. Vargas-Soler, Diego M. Barrera, Laura N. Palacio, Ulises Granados, Luis G. Uribe

**Affiliations:** 1 Universidad del Rosario, Bogotá, Colombia. Hospital Internacional de Colombia, Bucaramanga, Colombia. mairawm@hotmail.com Universidad del Rosario Universidad del Rosario Hospital Internacional de Colombia Bogotá Colombia mairawm@hotmail.com; 2 Fundación Cardiovascular de Colombia, Bucaramanga, Colombia. dorisquintero@fcv.org Fundación Cardiovascular de Colombia Bucaramanga Colombia dorisquintero@fcv.org; 3 Hospital Internacional de Colombia, Fundación Cardiovascular de Colombia, Bucaramanga, Colombia. josevargas@fcv.org Hospital Internacional de Colombia Fundación Cardiovascular de Colombia Bucaramanga Colombia josevargas@fcv.org; 4 Hospital Internacional de Colombia, Fundación Cardiovascular de Colombia, Bucaramanga, Colombia. diegobarrera@fcv.org Hospital Internacional de Colombia Fundación Cardiovascular de Colombia Bucaramanga Colombia diegobarrera@fcv.org; 5 Hospital Internacional de Colombia, Fundación Cardiovascular de Colombia, Bucaramanga, Colombia. laurapalacio@fcv.org Hospital Internacional de Colombia Fundación Cardiovascular de Colombia Bucaramanga Colombia laurapalacio@fcv.org; 6 Hospital Internacional de Colombia, Bucaramanga, Colombia. ulisesgranados@fcv.org Hospital Internacional de Colombia Bucaramanga Colombia ulisesgranados@fcv.org; 7 Hospital Internacional de Colombia, Fundación Cardiovascular de Colombia, Bucaramanga, Colombia. luisuribe@fcv.org Hospital Internacional de Colombia Fundación Cardiovascular de Colombia Bucaramanga Colombia luisuribe@fcv.org

**Keywords:** Healthcare Associated Infection, Antibiotic Resistance, Hospital Infection Control Services, COVID-19, Infecciones Asociadas con el Sistema de Salud, Resistencia a Antibióticos, Servicios de Control de Infección Hospitalaria, COVID-19, Infecções Associadas ao Sistema de Saúde, Resistência a Antibióticos, Serviços de Controle de Infecção Hospitalar, COVID-19

## Abstract

**Introduction:**

Healthcare-associated infections pose a significant challenge, contributing to hospital morbidity and mortality.

**Objective:**

To describe the behavior of Healthcare Associated Infections before and during the pandemic reported to a high-complexity health institution in Colombia.

**Material and Methods:**

In our retrospective observational study on Healthcare-Associated Infections (HAIs), we analyzed data from all in patients diagnosed with HAIs between 2018 and 2020. This included clinical, demographic, microbiological, and microbial susceptibility information collected from the Committee on Nosocomial Infections' prospective database. Data from 391 isolates were obtained using Whonet software for antimicrobial resistance surveillance.

**Results:**

We found 504 cases of HAIs (2018-2020) with an overall in-hospital infection rate of 2.55/1000 patient days. The median age for pediatric patients was 5 years, and for adults, 56 years, with 57% male. The leading admission diagnoses were oncologic disease complications (31%). Bacteremia had a 30-day mortality rate of 13%, predominantly catheter-associated (37%). Gram-negative bacilli, notably Klebsiella pneumoniae, Escherichia coli, and Pseudomonas aeruginosa, represented 58% cases of HAI.

**Discussion:**

The critical need for specific interventions and antimicrobial management to control HAIs, especially given the challenges posed by the COVID-19 pandemic, is highlighted.

**Conclusions:**

This is the first report on HAIs incidence at a tertiary hospital in Bucaramanga, Santander (Colombia). Bacteremia was predominant; 75% of HAIs patients had comorbidities. Gram-negative bacilli prevailed; a notable rise in ICU respiratory infections occurred during the 2020 COVID-19 pandemic. Resistance to cephalosporins and carbapenems was prevalent.

## Resumen

**Introducción:**

Las infecciones asociadas a la atención en salud (IAAS) representan un reto porque estas contribuyen a la morbilidad y mortalidad hospitalaria.

**Objetivo:**

Describir el comportamiento de las IAAS antes y durante la pandemia, las cuales fueron reportadas a una institución de salud de alta complejidad en Colombia.

**Materiales y métodos:**

En nuestro estudio observacional retrospectivo de las IAAS, analizamos los datos de todos los pacientes hospitalizados que fueron diagnosticados con una IAAS entre 2018 y 2020. Esto incluyó información clínica, demográfica, microbiológica y de susceptibilidad microbiana recabada de la base de datos prospectiva del Comité de Infecciones Nosocomiales. Los datos de 391 aislamientos se obtuvieron utilizando el programa informático Whonet para la vigilancia de la resistencia a los antimicrobianos.

**Resultados:**

Encontramos 504 casos de IAAS entre 2018 y 2020 con una tasa global de infección intrahospitalaria de 2,55/1000 pacientes al día. La mediana de edad de los pacientes pediátricos fue de 5 años y la de los adultos de 56 y el 57% de ellos eran varones. Los principales diagnósticos de ingreso fueron complicaciones oncológicas (31%). La bacteriemia tuvo una tasa de mortalidad a los 30 días del 13%, predominantemente asociada al uso de catéter (37%). Los bacilos gramnegativos, sobre todo Klebsiella pneumoniae, Escherichia coli y Pseudomonas aeruginosa, representaron el 58% de los casos de IAAS.

**Discusión:**

Se destaca la necesidad crítica de contar con intervenciones específicas y de gestión antimicrobiana para controlar las IAAS, especialmente teniendo en cuenta los retos que planteó la pandemia de Covid-19.

**Conclusiones:**

Este es el primer informe sobre la incidencia de las IAAS en un hospital terciario de Bucaramanga, Santander (Colombia). La bacteriemia predominó y 75% de los pacientes con IAAS presentaban comorbilidades. Predominaron los bacilos gramnegativos y se produjo un notable aumento de las infecciones respiratorias en las UCI durante la pandemia Covid-19 de 2020. Fue prevalente la resistencia a las cefalosporinas y a los carbapenémicos.

## Resumo

**Introdução:**

As infeções associadas aos cuidados de saúde representam um desafio significativo, contribuindo para a morbilidade e mortalidade hospitalar.

**Objetivo:**

Descrever o comportamento das Infecções Relacionadas à Assistência à Saúde antes e durante a pandemia notificadas a uma instituição de saúde de alta complexidade na Colômbia.

**Material e Métodos:**

Em nosso estudo observacional retrospectivo sobre Infecções Relacionadas à Assistência à Saúde (IRAS), analisamos dados de todos os pacientes internados com diagnóstico de IRAS entre 2018 e 2020. Isso incluiu informações clínicas, demográficas, microbiológicas e de suscetibilidade microbiana coletadas do Comitê no banco de dados prospectivo de infecções hospitalares. Os dados de 391 isolados foram obtidos utilizando o software Whonet para vigilância da resistência antimicrobiana.

**Resultados:**

Foram encontrados 504 casos de IRAS (2018-2020) com taxa global de infecção hospitalar de 2,55/1.000 pacientes-dia. A idade média para pacientes pediátricos foi de 5 anos, para adultos 56 anos, sendo 57% do sexo masculino. Os principais diagnósticos de admissão foram complicações de doenças oncológicas (31%). A bacteremia teve uma taxa de mortalidade em 30 dias de 13%, predominantemente associada ao cateter (37%). Bacilos Gram-negativos, notadamente Klebsiella pneumoniae, Escherichia coli e Pseudomonas aeruginosa, representaram 58% dos casos de IRAS.

**Discussão:**

É destacada a necessidade crítica de intervenções específicas e gestão antimicrobiana para controlar as IACS, especialmente tendo em conta os desafios colocados pela pandemia da COVID-19.

**Conclusões:**

Este é o primeiro relatório sobre a incidência de IRAS em um hospital terciário em Bucaramanga, Santander (Colômbia). A bacteremia foi predominante; 75% dos pacientes com IRAS apresentavam comorbidades. Prevaleceram bacilos Gram-negativos; um aumento notável nas infecções respiratórias em UTI ocorreu durante a pandemia de COVID-19 de 2020. A resistência à cefalosporina e aos carbapenêmicos foi prevalente.

## Introduction

Healthcare-Associated Infections (HAIs) continue to contribute substantially to hospital morbidity and mortality^1^. Especially when associated with antibiotic-resistant microorganisms. Prevalence studies in the United States (US) suggest that 30% of HAIs occur in intensive care units (ICUs)^2^^,^^3^. The increase in the incidence of HAIs is largely due to the multiple interventions of modern medicine, by using new medical devices, organ transplants, broad-spectrum antibiotics, and long hospital stays, among others^4^^,^^7^ These HAIs have become one of the most important challenges in medical practice, as pointed out by the World Health Organization (WHO)^8^.

It is estimated that the prevalence of HAIs in developing countries is significantly higher compared to developed countries, as described in a recent meta-analysis where regions such as North America and Europe report a prevalence between 4 to 7 cases per 100 hospitalized patients, contrary to reports in Latin America, Africa, and Asia where the prevalence can reach up to 16 cases per 100 hospitalized patients^9^. This situation is even more worrying when describing the incidence of HAIs in Intensive Care Units (ICU), where countries such as Brazil reach between 14-62 cases per 1000 patients compared to the United States which reports between 6-9 cases per 1000 patients^4^^,^^10^. This data coincides with estimates that between 20-30% of all HAIs in the hospital are acquired in ICUs with the presence of Multi-Drug Resistant Organisms (MDRO)^10^^,^^11^.

In Colombia, Enterobacterales (*Escherichia coli, Klebsiella* spp, and *Enterobacter* spp) rank highest in the epidemiology of HAIs (Healthcare-Associated Infections)^12^. These bacteria can develop resistance to various carbapenems through the production of hydrolytic enzymes such as Extended-Spectrum Beta-Lactamases (ESBLs), *AmpC* cephalosporinases, carbapenemases, or mutations in outer membrane proteins^13^. Carbapenemases were first reported in Colombia in 2006, and since then, various studies have demonstrated a significant increase in bacterial resistance. For instance, cephalosporins showed a resistance rate of 21.70% between 1997-2000, and subsequently, between 2013-2016, an increase of 63.00% was observed in the analyzed isolates^14^.

During the SARS-CoV-2 pandemic, hospital institutions faced immense pressure, demanding significant economic and human efforts, especially from healthcare workers. It required relocation and training in new hospital areas divided to treat both COVID and non-COVID patients, particularly in ICUs^15^.

Antimicrobial resistance during the SARS-CoV-2 pandemic, along with the increased use of antibiotics, raised the likelihood of developing a secondary HAI (Healthcare-Associated Infection) due to COVID-19^16^^,^^17^. The reason why active epidemiological surveillance of HAIs within healthcare institutions is necessary is to understand which microorganisms are most common and to observe changes in antimicrobial susceptibility profiles that may prevent future outbreaks within healthcare facilities^18^. The objective of this study is to describe the behavior of Healthcare Associated Infections before and during the pandemic reported to a high-complexity health institution in Colombia, between the years 2018 and 2020.

## Material and Methods

### Study design

This was a retrospective cohort study aimed at describing the changes in HAIs in the high complexity institution before and during the COVID-19 pandemic. The study was conducted over three years from January 1, 2018 to December 31, 2020, in which 244,889 hospital admissions were reported. We analyzed all patients admitted to the hospital from January 1, 2018 to March 29, 2020, the date on which the first SARS-CoV-2 positive patient was admitted to our institution (168,034 hospital admissions) and compared it with patients admitted during the COVID-19 pandemic that included the period of time between March 30, 2020 to December 31, 2020 (76,885 hospital admissions).

### Clinical setting and data collection

The study took place in a Joint Commission-accredited 286-bed fourth level care institution in Bucaramanga, Santander, Colombia. The hospital has a 187-bed capacity in adult general wards and 99 in adult intensive care unit (ICU). Case definition for HAI followed the Centers for Disease Control HAI criteria, which defines HAI as a localized or systemic condition resulting from an adverse reaction to the presence of an infectious agent(s) or its toxin(s), after the 3rd hospital day (day of hospital admission is day 1). There must be no evidence that the infection was present or incubating at the time of admission hospital^19^.

All patients with a confirmed diagnosis of HAIs (n=504) were included in the analysis. Clinical and demographic data were collected from the hospital's electronic medical records, including age, sex, date of admission, type of admission, discharge date, status of the patient at discharge, pre-existing comorbidities; antimicrobial therapy; diagnosed HAIs and microbiological cultures performed, locating the site of infection, date of HAI onset and microbiological confirmation. The analysis for site of infection was performed according to the following groups, bloodstream, includes bacteremia associated with central venous catheter, Hickman Broviac catheter, Mahurkar catheter, peripherally inserted central catheter, fungemia and bacteremia associated with mucosal barrier injury; urinary tract includes urinary tract and bladder catheter-associated infections; surgical site includes superficial and deep surgical site infection, organ space surgical site infection, and soft tissue infection; respiratory tract includes tracheobronchitis associated with mechanical ventilation, cases of pneumonia, tracheitis, aspiration pneumonia, and aspiration tracheobronchitis. For COVID-19 patients, laboratory confirmation of SARS- CoV-2 was defined as a positive result of real-time reverse transcriptase-polymerase chain reaction assay of nasal and pharyngeal swabs^20^.

### Microbiological identification

For each case of HAI, the type of infection, pathogen identification and microbiological susceptibility were obtained from the medical record and WHONET Software (version 5.6), used for surveillance of antimicrobial susceptibility^21^. Identification of microbial species and antimicrobial resistance patterns were determined using the VITEK-2 Compact system (BioMerieux SA, France). The following quality control strains were included: *Staphylococcus aureus* [American Type Culture Collection (ATCC) 25923], *Escherichia coli* (ATCC 25922), *Klebsiella pneumoniae* (ATCC 700603; BAA1705) and *Pseudomonas aeruginosa* (ATCC 27853).

### Data Set

The validated information was stored in GitLab^22^.

### Statistical analysis

Clinical and microbiological data were collected and by the principal investigator and data analyst. For statistical analyses, a descriptive analysis was initially performed, where categorical values are presented as proportions and continuous variables as means and standard deviation (SD). Otherwise, these variables were described as medians and their interquartile range (IR). The Chi2 test was used to determine if there were statistically significant differences between the categorical variables and the student’s t-test or the Mann-Whitney U-test for the continuous variables, according to their distribution.All data were analyzed in Stata statistical software version 15.0 (Stata Corporation, College Station, TX). Statistical significance was defined as a p-value <0.05.

### Ethics considerations

This study was approved by the ethics committee of the Fundación Cardiovascular de Colombia (Acta # 525 of December 15, 2020).

## Results

A total of 244,889 hospital admissions comprising 168,034 hospital admissions recorded from January 1, 2018, to March 29, 2020 (referred to as the 2018/2019 cohort) and 76,855 hospital admissions from March 30, 2020, to December 31, 2020 (described as the 2020 cohort). Among all the admissions, 504 cases of HAI were reported, with an average of 166.52 cases per year in the 2-year period prior to COVID-19 (total of 333 cases, rate of 0.13% per hospital admission), and 171 cases reported after the first case of SARS-CoV-2 infection was confirmed in the institution on March 29, 2020 (rate of 0.67% per hospital admission). A detailed description of the patient demographics is provided in (Table 1).

**Table 1:** Characteristics of the patients (n=504)

	2018/2019 (year)	2020 (year)	p-value
Patients	333	171	-
Observation time, person-days	114,776	83,087	-
Gender (male)	54.00 (180)	65.00 (111)	0.0181‡
Age, years (mean ± SD)	46.30 ± 28.0	48.60 ± 23.8	0.3594¥
Pediatric patients (<18 years)	25.22 (84)	16.95 (29)	0.0345‡
Age of pediatric patients, (mean ± SD)	4.40 ± 5.10	7.42 ± 6.20	0.3594¥
Age of adult patients, (mean ± SD)	56.80 ± 17.7	57.0 ± 15.9	0.9012¥
Coexisting conditions %(n)			
Coronary heart disease	10.81 (36)	11.11 (19)	0.9186‡
Chronic lung disease	8.10 (27)	15.78 (27)	0.0090‡
Autoimmune disease	3.60 (12)	5.26 (9)	0.3774‡
Endocrine disease	5.10 (17)	5.84 (10)	0.7258‡
Chronic renal disease	8.70 (29)	21.63 (37)	< 0.0001‡
Hypertension	34.53 (115)	34.50 (59)	-
Diabetes mellitus	12.91 (43)	25.73 (44)	0.1362‡
Obesity	3.00 (10)	14.61 (25)	< 0.0001‡
Psychiatric disorder	19.82 (66)	15.78 (27)	0.0540‡
Cancer	45.64 (152)	19.88 (34)	<0.00001‡
Genetic disease	2.10 (7)	7.01 (12)	0.0061‡
Central nervous system disorders	18.31 (61)	39.76 (68)	< 0.0001‡
Immunosuppression	28.82 (96)	35.67 (61)	0.1188‡
Infection site. %(n)			
Bloodstream	42.94 (143)	47.36 (81)	0.3470‡
Urinary tract	16.51 (55)	23.97 (41)	0.0452‡
Surgical site	27.32 (91)	12.86 (22)	0.0002‡
Respiratory tract	13.21 (44)	15.78 (27)	0.4449‡
Length of hospital stay (days), median (IQR)	77 (18-95)	33 (17-50)	0.4278¥
Admission to ICU	36.33 (121)	52.63 (90)	0.0004‡
Central venous catheter	35.13 (117)	38.59 (66)	0.5046‡
Urinary catheter	19.81 (66)	25.14 (43)	0.1113‡
Invasive ventilation	15.91 (53)	20.46 (35)	0.2025‡
Positive RT-PCR for SARS-CoV-2	N/A	33.91 (58)	-
Mortality. %(n)	9.90 (33)	28.07 (48)	< 0.0001‡
Mortality rate (95% CI) per 1000 patient-days	2.90 (2.01- 3.99)	5.77 (4.30- 7.59)	-
30-day mortality in patients with bacteremia. %(n)	5.10 (17)	11.11 (19)	0.0133‡
Re-admission. %(n)	16.81 (56)	17.54 (30)	0.8433‡

Notes: % percentage; IQR: interquartile range; SD: standard deviation; ‡p value determined by Chi2 test; ¥p value determined by Student's t test.

In both cohorts, most of the patients were men, especially in the 2020 cohort (65.00%). The mean age was similar between cohorts (46.3 vs 48.6 years), but the proportion of pediatric patients was higher in the 2018/2019 cohort (25.22%). In the 2018/2019, cancer, immunodeficiencies, and hypertension were the most common coexisting conditions, compared to the 2020 cohort where central nervous system (CNS) pathologies, immunodeficiencies, and hypertension were the most common. In the 2020, there was a 21.45%, 12.93%, and 11.61% increase in patients with HAIs that also presented with CNS pathologies, chronic renal disease, and obesity, respectively, compared to the 2018/2019 cohort (p < 0.0001). Conversely, there was a 25.76% reduction in the 2020 cohort, compared to the 2018/2019 cohort, among patients with cancer and HAIs. In both cohorts, the more commonly identified site of infection was blood. However, in the 2020 cohort there was a significant reduction in surgical site infections (p = 0.0002), likely due to the decreased surgical procedures performed in our institution during the early stages of the COVID-19 pandemic. While the median length of stay decreased between the 2018/2019 cohort (31 days) and the 2020 cohort (28 days), the proportion of patients admitted to the ICU increased by 16.30%. As for invasive devices, patients hospitalized in 2020 were more likely to have urinary catheters and respiratory support through mechanical ventilation. Overall mortality was significantly higher in the 2020 cohort (28.00%), with a mortality rate of 5.77 (95% CI 4.3-7.6) per 1,000 patients-days. By contrast, the mortality rate in 2018/2019 was 2.9 (95% CI 2.0-4.0) per 1,000 patients- days. Rates of readmission were similar between both cohorts (16.81% vs 17.54%, p = 0.84). Within the cases observed in the 2020 cohort, 58 HAIs (33.91%) corresponded to patients who tested positive for SARS-CoV-2. These patients were more likely to be male, older (median age 60.7 years) and obese (Supplementary Table 1). A higher proportion of the patients with both SARS-CoV-2 infection and HAIs died (63.70%) compared to those without SARS-CoV-2 (12.52%). However, the mortality rate was similar between subgroups.

**Figure 1:**
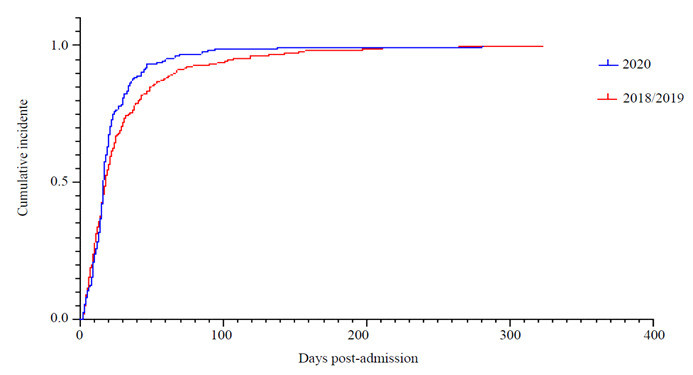
Cumulative incidence of HAIs

While the overall proportion of patients with HAIs was similar between cohorts, patients with HAIs in the 2020 cohort were more likely to become infected earlier after being admitted to the hospital (Figure 1).

**Figure 2:**
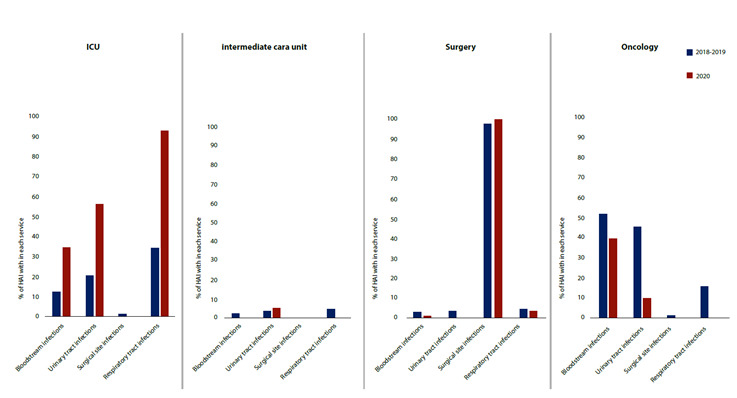
Frequency of HAIs per site of infection and hospitalization service

The frequency of HAIs by site of infection and hospitalization service was similar in most cases, except for bloodstream infections, urinary tract infections, and respiratory tract infections of patients in intensive care unit, which increased to >100% for respiratory infections in the 2020 cohort, according to the significant increase in the 30.00% available beds ICU in the number of COVID-19 cases seen (Figure 2, Supplementary Table 2).

Most infections were monomicrobial 486 (96.42%), only 27 infections were polymicrobial, of which 17 were gram-negative pathogen and 8 with one gram-positive and one gram-negative organism. There was one mixed infection with two fungi and another infection with gram-negative organism and one fungus. In both cohorts, the most common isolations were Klebsiella pneumoniae, Escherichia coli and Pseudomonas aeruginosa (Table 2). Overall, Enterobacterales were the causative organisms in 52.54% of HAIs in the 2018/2019 cohort and 50.28% of HAIs in the 2020 cohort. In the 2020 cohort, there was a 4.65% increase in cases of Pseudomonas aeruginosa compared to the prior years. Similarly, infections by Candida spp. Increased by 5.78%. A detailed distribution of causative organism per site of infection is provided in (supplementary Figure 1).

While Enterobacterales were the most common organism isolated from bloodstream, urinary tract and surgical site HAIs, Pseudomonas aeruginosa was the most common pathogen in respiratory tract HAIs.

**Table 2:** Microorganisms isolated.

Microorganism	2018/2019 cohort %(n)	2020 cohort %(n)	% change
Klebsiella pneumoniae	24.62 (82)	21.64 (37)	-2.99
Escherichia coli	13.51 (45)	15.20 (26)	1.69
Pseudomonas aeruginosa	12.31 (41)	16.96 (29)	4.65
Staphylococcus aureus	6.31 (21)	8.77 (15)	2.46
Enterobacter cloacae	5.41 (18)	3.51 (6)	-1.9
Proteus mirabilis	4.20 (14)	1.75 (3)	-2.45
Acinetobacter baumannii	2.10 (7)	1.75 (3)	-0.35
Staphylococcus epidermidis	1.80 (6)	2.34 (4)	0.54
Pseudomonas putida	1.50 (5)	1.17 (2)	-0.33
Morganella morgannii	1.50 (5)	0.58 (1)	-0.92
Aeromonas hydrophila	1.20 (4)	0.58 (1)	-0.62
Serratia marcescens	1.20 (4)	1.75 (3)	0.55
Enterobacter aerogenes	1.20 (4)	1.17 (2)	-0.03
Streptococcus mitis	0.90 (3)	0.00 (0)	-0.90
Streptococcus agalactiae	0.60 (2)	0.00 (0)	-0.60
Stenotrophomonas maltophilia	0.90 (3)	0.58 (1)	-0.32
Candida albicans	0.60 (2)	3.51 (6)	2.91
Candida tropicalis	0.30 (1)	2.92 (5)	2.62
Candida parapsilosis	0.90 (3)	1.17 (2)	0.27
Candida glabrata	0.30 (1)	0.00 (0)	-0.30
Candida haemulonii	0.00 (0)	0.58 (1)	0.58
Candida krusei	0.30 (1)	0.00 (0)	-0.30
Enterococcus faecalis	0.60 (2)	3.51 (6)	2.91
Enterococcus faecium	0.00 (0)	1.17 (2)	1.17
Proteus penneri	0.60 (2)	0.00 (0)	-0.60
Klebsiella oxytoca	0.30 (1)	0.00 (0)	-0.30
Pantoea spp	0.90 (3)	0.00 (0)	-0.90
Staphylococcus capitis	0.30 (1)	0.00 (0)	-0.30
Staphylococcus warneri	0.30 (1)	0.00 (0)	-0.30
Staphylococcus hominis	0.00 (0)	0.58 (1)	0.58
Streptococcus anginosus	0.00 (0)	0.58 (1)	0.58
Streptococcus sanguinis	0.00 (0)	0.58 (1)	0.58
Streptococcus pseudoporcinus	0.00 (0)	0.58 (1)	0.58
Aeromonas sobria	0.30 (1)	0.00 (0)	-0.30
Salmonella enterica arizonae	0.30 (1)	0.00 (0)	-0.30
Haemophilus influenzae	0.30 (1)	0.00 (0)	-0.30
Achromobacter xylosoxidans	0.30 (1)	0.00 (0)	-0.30
Myroides spp	0.30 (1)	0.00 (0)	-0.30
Listeria monocytogenes	0.30 (1)	0.00 (0)	-0.30
Burkholderia cepacia	0.00 (0)	0.58 (1)	0.58
Citrobacter koseri	0.00 (0)	0.58 (1)	0.58
Citrobacter youngae	0.00 (0)	0.58 (1)	0.58
Cupriavidus pauculus	0.00 (0)	0.58 (1)	0.58
Negative	1.80 (6)	0.58 (1)	-1.22
Without germ	11.41 (38)	4.09 (7)	-7.32

**Figure 3:**
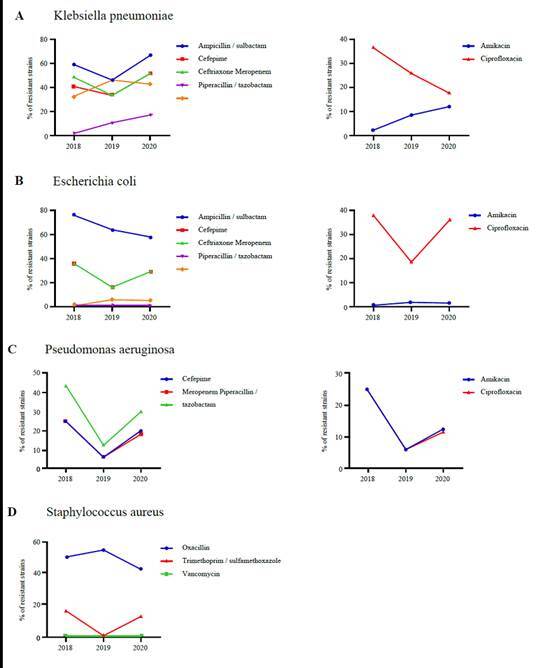
Comparison of antimicrobial resistance over time

Given that *Klebsiella pneumoniae, Escherichia coli, Pseudomonas aeruginosa*, and S*taphylococcus aureus* represent 56.75% of all cases identified as HAIs among the patients included in this study, we characterized the antimicrobial susceptibility patterns from the isolates obtained between 2018 and 2020 (Figure 3). During this period the percentage of extended spectrum beta-lactamase (ESBL)- producing *Escherichia coli* and *Klebsiella pneumoniae* remained at approximately 37.00% and 45.00%, respectively. A progressive increase in resistance to meropenem was observed in *Klebsiella pneumoniae*, from 1.70% in 2018 to 17.00% in 2020.

Bacteremia was the most common type of HAI. In the univariate analysis (see Table 3), statistical differences were observed between the living and dead of those who had had a previous infectious disease compared to those who had not had previous infections. In addition, patients diagnosed with SARS-CoV-2 showed statistical differences in mortality and hospital stay was short for patients who died of bacteremia and pediatric population.

**Table 3:** Univariate analysis 30-day mortality (224 bacteremia events)

Variables	Alive (n=193) %(n)	Dead (n=31) %(n)	p-value
Years			0.080†
2018	15.54 (30)	22.58 (7)	
2019	36.26 (70)	16.12 (5)	
2020	48.18 (93)	61.29 (19)	
Gender			0.815‡
Female	40.93 (79)	38.70 (12)	
Masculine	59.06 (114)	61.29 (19)	
Population Group			0.012‡
Pediatrics	35.75 (69)	12.90 (4)	
Adult	64.24 (124)	87.09 (27)	
Age pediatric group, years (mean ± SD)	5.40 ± 3.80	1.90 ± 1.03	0.9205¥
Age adult group, years (mean ± SD)	52.70 ± 24.50	61.50 ± 30.50	0.8320¥
Comorbidities in adult	(n=124)	(n=27)	
Oncologic disease	51.61 (64)	44.44 (12)	0.6436‡
Coronary artery disease	14.51 (18)	18.51 (5)	0.070‡
Chronic lung disease	12.90 (16)	11.11 (3)	0.510‡
Hormonal disease	4.83 (6)	7.40 (2)	0.169‡
Chronic renal disease	16.12 (20)	18.51 (5)	0.834‡
Hypertension	41.93 (52)	29.62 (8)	0.657‡
Diabetes mellitus	20.16 (25)	22.22 (6)	0.275‡
Obesity	10.48 (13)	11.11 (3)	0.529‡
Psychiatric disorder	25.80 (32)	40.74 (11)	0.066‡
Comorbidities in Pediatrics	(n=69)	(n=4)	
Oncologic Disease	47.82 (33)	75.00 (3)	0.111‡
Gastrointestinal Disease	36.23 (25)	25.00 (1)	0.996‡
Genetic Disease	15.94 (11)	25.00 (1)	0.083‡
Cardiovascular Disease	4.34 (3)	0.00 (0)	-
Previous infectious diseases	14.49 (10)	25.00 (1)	0.063‡
Immune suppression all ages	52.84 (102)	41.93 (13)	0.259‡
Diagnostic SARS-CoV 2, year 2020 (n: 112)	11 (4.74)	10 (27.00)	<0.0001‡
Length of hospital stay, days (mean ± SD)	74.8 ± 85.50	31.8 ± 23.00	0.0002¥
Adequate empiric therapy	68.39 (132)	55.88 (19)	0.4335‡

SD: Standard Deviation; Hospital stays; was defined as the total number of days the patient was in the hospital until discharge; Immune suppression: Neutropenia, chemotherapy, malnutrition, and HIV/AIDS. Psychiatric disorder: Epilepsy, delirium, depression, schizophrenia, bipolar affective disorder, acute psychotic, and schizophrenia. †: determined by Fischer's exact test ‡: p value determined by Chi2 test; ¥: p value determined by Student’s t test.

## Discussion

In this study, we describe for the first time the overall incidence rate of healthcare-associated infections of HAI is 2.55 patients per 1,000 days. This is the first report of the incidence rate of HAI in a third level hospital in the city of Bucaramanga, Santander (Colombia). In Latin America there is little data on the burden of HAI. However, some countries have made progress in the characterization of this problem, as described in the study of Prevalence of Adverse Events in Latin American Hospitals - IBEAS in which nosocomial infections or currently known as HAI was the most frequent event with 37.00% in agreement, the result for Colombia occupied the first place, followed by other events related to procedures and care^23^.

Our study revealed that bloodstream infections were the most frequent, accounting for 45.05% of the HAIs identified during the last 3 years; we observed that this frequency increases significantly in patients with immunosuppression or oncologic disease. As reported by other studies in Latin America^24^. The dynamics of nosocomial infection sites may change according to the use of new medical devices and the implementation of immunosuppressive therapies implemented by each hospital institution^25^.

The most common microorganisms for the different types of infections were Gram-negative bacilli (Klebsiella pneumoniae, Escherichia coli and Pseudomonas aeruginosa). In the case of Gram-positives it was Staphylococcus aureus. Similar data have been reported from other institutions of equal complexity where Gram-negative bacilli are the most common microorganisms associated with nosocomial infections^26^^,^^28^.

Another important finding was that 75.20% of patients with HAI had some comorbidities: arterial hypertension 34.50%, psychiatric disease 17.79%, diabetes 19.32%, chronic renal disease 15.16%, chronic pulmonary disease and coronary disease in 10.96% of the cases analyzed. Comorbidities being a considerable risk factor, which can contribute to long hospital stays, generating a negative impact on healthcare systems^29^.

As reported by several studies, bacteremia is associated with high mortality. In fact, (13/74) 18.00%, several studies have demonstrated similarly dismal outcomes in immunosuppressed or critically ill patients who develop sepsis, (1/14) including liver transplant patients with gram-negative sepsis^30^^,^^31^.

A comparison of antimicrobial resistance over time showed a significant increase in resistance to cephalosporins from 22.00% to 47.00% for Enterobacteriaceae. On the contrary, resistance to ciprofloxacin remained between 16% and 20% during the last 3 years; as for piperacillin tazobactam, there were no significant differences with a resistance between 7% and 8%. Contrary to other studies where the highest bacterial resistance is associated with cephalosporins and carbapenems^32^. The number of Pseudomonas aeruginosa isolates in HAI increased from 7 reported for 2018 to 43 by 2020, this significant increase may be attributed possible outbreaks that occurred in the ICU, increased use of medical devices in patients with COVID-19 among others.

The multidrug-resistant (MDR) organisms comprised 15.10% of the total isolates, including BLEE, CRE and MDR *Pseudomonas* MDR infections. Overall, a significant increase in reported BLEE was observed by 2020. This can be attributed to the expansion of the hospital capacity installed by COVID-19, the reconversion of services was carried out, increasing the number of intensive care beds from 21 beds to 51 beds for adults and temporarily opening in the Special Registry of Health Providers - REPS, 17 beds for adult intensive care and 18 beds for adult intermediate care; with the consequent growth in human talent, equipment and biomedical devices.

The empiric antibiotic most used in the health institution was piperacillin/tazobactam, used in 40.00% of patients with HAIs; no significant increase in resistance to this type of antibiotic was observed in our analysis. Similar data have been reported by other hospital institutions^31^. During the development of this study, information from both pediatric and adult populations was consolidated to perform a comprehensive and detailed analysis of all HAIs detected during the last 3 years in our institution.

It is important to mention that during the hospital stay, the causative agent was identified for 90% of all the detected HAIs together with the antimicrobial susceptibility profile, obtaining valuable information that allowed the treating physician to implement the appropriate antibiotic therapy. However, it is important to point out that a small percentage of the cultures performed could not identify the causal agent of the infection, a situation that may be associated with the fact that the high complexity institution is a reference health institution where a high number of patients are referred by other health institutions of lower complexity where patients have received empirical antibiotic treatment, which in some cases does not allow isolating and identifying the microorganism that is causing the infection for 100% of the cultures performed in the institution.

Urinary tract infections accounted for 20.00% to 56.10% of HAIs in ICU, general hospitalization, and oncology services, 46.05% of HAIs being associated with bladder catheter use, making bladder catheter use a risk factor, as described in other studies. Recent studies have shown that the inadequate use of antibiotic prophylaxis in urinary tract infections outweighs the benefits and contributes to the increase of bacterial resistance^33^^,^^34^, for this reason it is only recommended to use antibiotics in symptomatic infections in catheterized patients and not to use prophylaxis after catheter change in the absence of symptoms of infection^35^.

Among the difficulties and limitations of the study, some stand out, such as the collection of all the relevant clinical information for the 504 patients with HAIs. A possible poor quality of some of the data collected in the clinical history may underestimate important information for the analysis. However, all the information collected was done together with the medical team to clarify any doubts or uncertainties that arose in the collection of these data.

Hospital-acquired infections contribute to prolonged hospital stays and present a substantial economic burden to healthcare systems. Studies show that middle-income countries experience a higher burden of HAIs compared to developed countries, so implementing measures to prevent HAIs in middle income countries appears to be a high-value information resource that can contribute to improved HAI control and surveillance. In addition, it can contribute to improve the standardization of reporting for the control of these infections^36^^,^^38^.

It is important to highlight the work performed by the Hospital Infection Committee, which collects, updates, and constantly informs all health personnel about the behavior of HAIs in this hospital. Simultaneously, it takes the measures required for any area of the hospital and highlights the importance of prevention and surveillance of these infections in the health system.

HAIs are not exclusive to any one hospital area, but some factors that contribute to the risk of contracting them have been described, whether due to long hospital stays, immunosuppression, surgical interventions, or the use of invasive medical devices, among others. For this reason, it is recommended that antimicrobial stewardship programs be implemented to effectively reduce the inappropriate use of antibiotics, especially in ICUs with the highest number of MDR organisms^39^^,^^40^.

## Conclusions

In conclusion, bacteremia was the most frequent infection in the total number of HAIs. The most common microorganisms were Gram-negative bacteria; no differences were found between the type of infection or hospital area analyzed. Resistance to piperacillin/tazobactam increased substantially in the last year for both Enterobacterales and *Pseudomonas*, as did oxacillin resistance for *Staphylococcus aureus*. Despite the increase in antimicrobial resistance, most patients received adequate empirical antimicrobial therapy, a significant increase in the number of respiratory infections identified in the ICU was observed for the 2020 cohort (during COVID-19), due to the high number of patients treated in the hospital for SARS-CoV-2.

It is suggested that empirical treatment regimens be based on specific data from epidemiological surveillance, analysis, and interpretation of HAIs in each hospital.

It is necessary to conduct research that allows defining the cost-benefit relationship for the prevention and control of HAIs, especially evaluating the long-term impact of biosafety protocols employed during the pandemic and the elevated use of certain broad-spectrum antibiotics in patients who presented COVID-19-related complications, and their relationship with the epidemiology of HAIs.
